# Research into the Applications of a Multi-Scale Feature Fusion Model in the Recognition of Abnormal Human Behavior

**DOI:** 10.3390/s24155064

**Published:** 2024-08-05

**Authors:** Congcong Li, Yifan Li, Bin Wang, Yuting Zhang

**Affiliations:** 1 School of Information Science and Technology, Hebei Agricultural University, Baoding 071001, China; xxzyt@hebau.edu.cn; 2 Hebei Key Laboratory of Agricultural Big Data, Baoding 071001, China; 20227060828@pgs0.hebau.edu.cn (Y.L.); 20227060847@pgs0.hebau.edu.cn (B.W.)

**Keywords:** abnormal behavior recognition, multi-scale convolution, dilated convolution, feature fusion, attention mechanism

## Abstract

Due to the increasing severity of aging populations in modern society, the accurate and timely identification of, and responses to, sudden abnormal behaviors of the elderly have become an urgent and important issue. In the current research on computer vision-based abnormal behavior recognition, most algorithms have shown poor generalization and recognition abilities in practical applications, as well as issues with recognizing single actions. To address these problems, an MSCS–DenseNet–LSTM model based on a multi-scale attention mechanism is proposed. This model integrates the MSCS (Multi-Scale Convolutional Structure) module into the initial convolutional layer of the DenseNet model to form a multi-scale convolution structure. It introduces the improved Inception X module into the Dense Block to form an Inception Dense structure, and gradually performs feature fusion through each Dense Block module. The CBAM attention mechanism module is added to the dual-layer LSTM to enhance the model’s generalization ability while ensuring the accurate recognition of abnormal actions. Furthermore, to address the issue of single-action abnormal behavior datasets, the RGB image dataset RIDS (RGB image dataset) and the contour image dataset CIDS (contour image dataset) containing various abnormal behaviors were constructed. The experimental results validate that the proposed MSCS–DenseNet–LSTM model achieved an accuracy, sensitivity, and specificity of 98.80%, 98.75%, and 98.82% on the two datasets, and 98.30%, 98.28%, and 98.38%, respectively.

## 1. Introduction

As China faces the increasing challenge of an aging population, new pressures emerge for social development. According to data from the seventh national census [1], there are 264.02 million people aged 60 and above in China, making up 18.70% of the total population. The Ministry of Civil Affairs predicts that during the 14th Five-Year Plan period, the elderly population will exceed 300 million, transitioning from mild to moderate aging. Thus, addressing the care of elderly people has become crucial [2]. Currently, most elderly people’s care options focus on home and community settings, with about 90% choosing to age at home [3]. Both home and community care face risks, as sudden illnesses caused by abnormal behaviors may go unnoticed, leading to injury or death. Therefore, ensuring the accurate and timely identification of unusual behaviors in elderly individuals living alone is essential.

A World Health Organization (WHO) [4] report indicates that four abnormal behaviors—falling, twitching, dizziness, and abnormal bending behavior (with vomiting as the main symptom)—occur more frequently and pose greater dangers among elderly individuals living alone. Approximately 684,000 people die each year globally due to falls, with the highest proportion of fatal injuries among those aged 60 and above. Twitching issues, stemming from conditions such as epilepsy, affect around 50 million people worldwide, with a significant number being older adults. Dizziness and vomiting in the elderly primarily result from cardiovascular diseases. Furthermore, among the 17 million annual deaths from non-communicable illnesses, 37% arise due to cardiovascular conditions. To address the subsequent risks posed by these behaviors, researchers domestically and internationally have pursued studies on behavior anomaly recognition algorithms [5,6,7,8]. This aims to accurately and promptly identify abnormal behaviors in elderly individuals living alone.

Currently, the identification and detection of anomalous behavior is primarily divided into unimodal and multimodal recognition. Multimodal recognition integrates various homogeneous or heterogeneous data through three distinct fusion methods [9]: data-level [10], feature-level [11], and decision-level [12]. However, unimodal recognition mainly consists of three approaches: those based on computer vision [13], environmental sensors [14], and wearable devices [15]. Methods based on computer vision leverage images or videos to extract higher-level features from data more effectively [16,17], allowing for a better description and understanding of behavioral information in scenes. Moreover, these methods do not require users to wear additional devices, ensuring user comfort while minimizing physiological harm from anomalous behavior and the subsequent medical costs [18]. Therefore, this study focuses on methods in the field of unimodal anomalous behavior recognition based on computer vision.

## 2. Related Work

In computer vision-based abnormal behavior recognition research, the most commonly used data types include RGB images, contour images, and skeleton point images. In studies using RGB image data for abnormal behavior recognition, Ding Xueqin [5] and others segmented the long-term structure of video sequences for modeling and used ResNets and BN-Inception X as the base networks to extract more motion features, achieving a recognition accuracy of 92.3%. She Jinshuo [19] and others addressed the challenge of complex spatial backgrounds with traditional algorithms by proposing a model that combines convolutional neural networks with bidirectional Long Short-Term Memory Networks (CNN-BiLSTMs) for feature fusion, achieving an accuracy of 94.6%. Cao Xugang et al. [20] proposed a method using a 3D-CNN model to extract spatiotemporal features from preprocessed TUG video sequences, enabling feature fusion at different spatiotemporal scales, ultimately achieving a feature classification accuracy of 92.2%. Li Chunhua et al. [21] addressed issues in abnormal behavior recognition models such as missed detections and poor robustness when abnormal behavior occurs by replacing the 3 × 3 convolutional blocks in YOLOv5s with an improved RepVGG module, optimizing the loss function, and achieving an average accuracy of 96.28%. Wang Xinwen et al. [22] proposed a Double 3D Residual Network (D3D) to address the issues of vanishing gradients and overfitting in complex backgrounds. By nesting residual networks within a residual network and effectively blending visual features from shallow and deep layers, the model achieved an accuracy of 99.2%. Jia Zhichao et al. [23] introduced a human action recognition algorithm based on global frequency domain pooling to tackle information loss and overfitting problems in 3D-ConvNet models for action recognition. By incorporating multiple low-frequency components to enrich the diversity of feature information after downsampling, the accuracy reached 63.0%. In the study of abnormal behavior recognition using silhouette data, Yang Peng [13] and others addressed the GaitSet algorithm’s problems of poor learning and classification capabilities. They proposed a multi-feature fusion convolutional network, MFFC-GaitSet, utilizing morphological processing to repair silhouette images and achieving a classification accuracy of 96.2%. Chen Wanzhi [6] and others tackled the issue of low recognition accuracy in traditional silhouette-based behavior recognition methods due to limitations in feature and model feature extraction capabilities. They introduced an improved GaitSet recognition algorithm that combines silhouette enhancement and attention mechanisms, achieving an accuracy of 88.1%. Chao et al. [24] proposed a gait recognition method based on deep sets, extracting spatial features directly from silhouette images using convolutional networks and performing deep sets on spatial features using compression operations on the time axis, with an average accuracy of 96.1%. Huang et al. [25] proposed a dual-branch action recognition model, Part3D, which included global and local branches. A three-dimensional convolution module (E3D) was designed to extract local and global behavioral features separately, achieving an accuracy of 97.8% in behavior recognition based on contour images. In the study of abnormal behavior recognition using skeletal point image data, Wang Xin et al. [7] applied the MOpenPose model to extract key skeletal features of the human body. They used a MobileNetV3 model with added residual structures for human behavior recognition, achieving an accuracy of 99.0%. Huang Xiaoyong et al. [26] proposed a tree-structured skeletal image and spatiotemporal block convolutional neural network model for fall detection. They utilized a three-dimensional posture estimation algorithm to obtain human joint nodes and then used the STB-CNN model, achieving an accuracy of 98.33%. Liang Ruiyan et al. [8] used the RPEpose model as a human key point detection model and introduced the X-Joint attention mechanism for abnormal behavior recognition classification, achieving an accuracy of 87.2%. The recognition of abnormal human behavior using skeletal point images relies too heavily on accurate posture estimation, is heavily influenced by external environmental factors, and provides only the position information of human joint nodes, lacking rich descriptions of human body morphology details. This limits the ability to capture specific details of certain behaviors, thereby reducing recognition accuracy. Behavior recognition based on contour images has low resolution requirements and effectively extracts behavioral features. Information captured in RGB images is more intuitive and easier to understand, containing rich color and texture information, enabling the capture of subtle motion features in human behavior. Therefore, this study selects RGB images and contour images to construct a dataset on abnormal human behavior.

This article improves the model by using a DenseNet–LSTM network as the model’s basic architecture foundation, in which DenseNet can effectively extract spatial information from images and LSTM can capture temporal dependencies in sequential data. By observing the activation of the convolutional layers in DenseNet and the gate units in LSTM, a better understanding of the decision-making process of the model in anomaly behavior recognition can be achieved, showing good interpretability [27]. However, using DenseNet–LSTM as the basic framework for anomaly behavior recognition also has its flaws. Therefore, this article makes the following improvements to the model:

(1) To address the issue of insufficient feature extraction in the initial convolution module of the DenseNet model structure, a self-built MSCS module was introduced. This not only increases the network width and adaptability to scale but also helps the model capture features at different levels of images. The parallel structure of the MSCS convolution increases the computational efficiency of the model, speeding up the feature extraction process.
(2) The 3 × 3 convolution in the DenseNet block structure is only able to extract features at a fixed scale, and as the depth of the model network increases, the 3 × 3 convolution brings more parameters, increasing training computational costs. To address these issues, this study constructed an Inception Dense structure to reduce the number of model parameters, decrease model complexity, and improve computational efficiency.
(3) To address the poor performance and weak generalization ability of the DenseNet–LSTM model on unseen datasets, this study introduced the CBAM attention mechanism into the LSTM structure. This helps reduce the network’s interference from background noise and enhances the model’s spatial and channel attention, better capturing the correlation and dependency between data.

In the final stage, the MSCS–DenseNet–LSTM model was thoroughly trained and tested using the self-built RIDS and CIDS datasets, as well as public datasets. A series of ablation and comparative experiments were designed, demonstrating that the model proposed in this study achieved significant improvements in recognition efficiency and generalization ability.

## 3. Materials and Methods

### 3.1. Selection of Abnormal Behavior Types

Elderly individuals living alone exhibit varied abnormal behaviors due to differing health conditions. This experiment recorded the four most prevalent types of abnormal behaviors: falling, twitching, dizziness, and vomiting. Each type was further categorized into specific abrupt scenarios; however, all these events were classified as one of the four main behaviors during abnormal behavior recognition and confusion matrix analysis. The classification of anomalous behavioral actions can be found in Table 1 below.

This study collected data on four types of fall behaviors. These included a simulation of an elderly individual suddenly falling while walking and the potential for secondary falls occurring during the process of getting back up. This categorization addresses the diversity and complexity of fall incidents, allowing for a more accurate identification and analysis of falls and their consequences. Additionally, this study examined four types of seizure actions in elderly individuals, simulating tonic seizures and cramp-like spasms in both sitting and lying positions. This classification aids in distinguishing different seizure types, which may bear distinct medical significance and management approaches in clinical practice. Furthermore, this research involved the collection of two types of dizziness actions. These events simulated instances where an elderly person experiences dizziness suddenly while walking or resting in a seated position. Given that dizziness is a common health concern among older adults, timely identification and intervention are crucial. Lastly, this study also included the collection of a vomiting action, simulating an elderly individual suddenly vomiting while bending over in a standing position. This scenario may reflect health challenges faced by seniors in specific environments, such as reactions due to fainting or dizziness.

These comprehensive categorization and simulation methods aid this paper in analyzing and understanding the mechanisms, identification techniques, and corresponding emergency responses of various abnormal behaviors more precisely. At the same time, these methods assist the model in better learning the characteristics of each anomalous behavior, enhancing its robustness in recognizing such behaviors, and thereby improving its accuracy in distinguishing various types of anomalies. Through the study of these data, we can gain a better understanding of the onset of different abnormal behaviors. This knowledge will enrich the design and improvement of future elderly behavior recognition models and contribute to explaining possible errors and displays in the confusion matrix.

**Figure 1:**
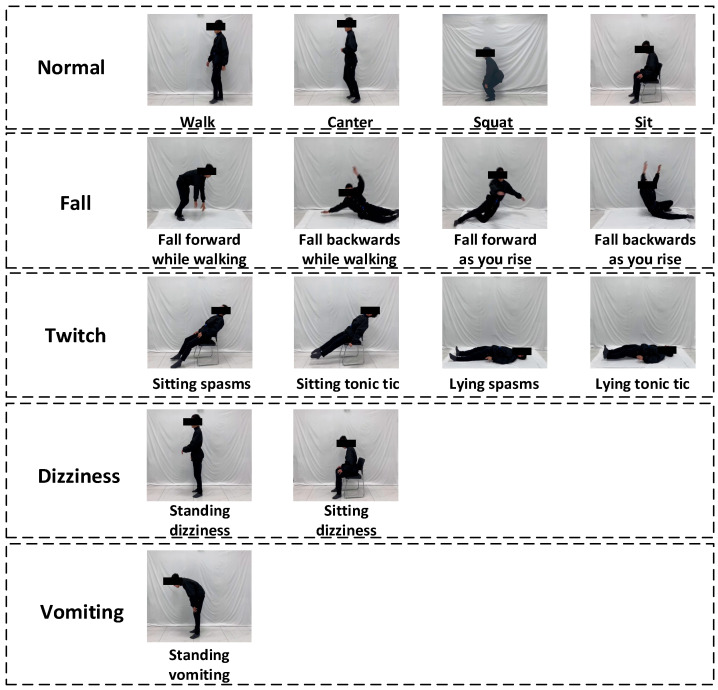
Simulations of various behavioral actions.

The analysis also included comparisons between four routine behaviors—slow walking, cantering, sitting, and squatting—and the identified abnormal behaviors. Figure 1 illustrates the simulation of each action.

### 3.2. Experimental Plan

**Figure 2:**
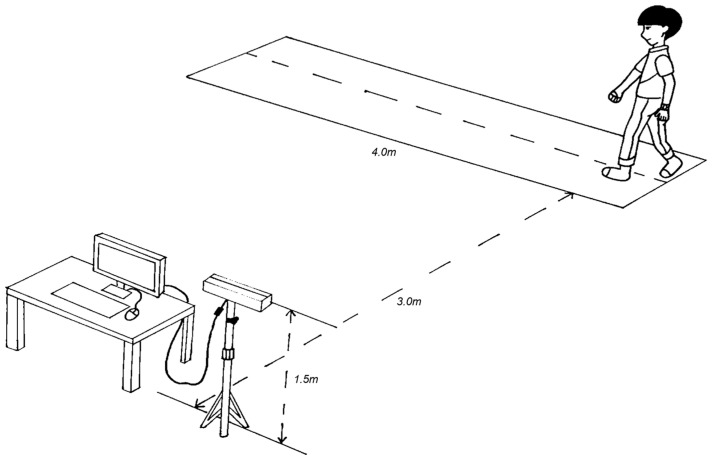
Experimental setup diagram.

This experiment utilized a Kinect 2.0 camera, boasting a pixel resolution of 1080P and stable performance, to capture video from a lateral perspective of various behavioral actions. The camera was mounted on a fixed tripod, set at a height of 1.5 m, with subjects positioned three meters away. Actions were simulated within a span of four meters, as illustrated in Figure 2. Given the unique challenges of detecting abnormal behaviors, collecting authentic data on elderly individuals proved difficult. Therefore, this study employed young participants dressed in elder-life simulation suits to mimic abnormal actions representative of the elderly. This experiment took place in a high-visibility environment, free of obstacles that might obscure the subjects, ensuring optimal operational conditions for the model and minimizing external noise interference on the results. A total of 25 individuals participated in this simulation, including 17 males and 8 females, with an average age ranging from 22 to 27 years. Soft mats surrounded the active area to prevent any injuries among participants, all of whom provided informed consent before participating in the experiment.

### 3.3. Experimental Preprocessing

Preprocessing of the RIDS dataset involved dividing the videos captured by the Kinect2.0 camera into individual action segments required for the experiment, each lasting 1–3 s. To facilitate model training and testing, as well as to reduce redundant information between consecutive frames, each video segment was converted into an image sequence based on a sampling rate of 30 frames per second. Subsequently, data augmentation was performed [28], where the images sampled from the videos were cropped around the center to obtain RGB image sequences of size 256 × 256 pixels.

For preprocessing the CIDS dataset, in order to minimize external factors such as background interference with the quality of silhouette image segmentation, the RGB image sequences were automatically cropped using the YOLOv5 [29] object detection algorithm to focus on the human body regions. Post-cropping, the silhouette images often contained numerous holes and erroneously connected regions, and thus morphological operations such as opening and closing [30] were introduced from image processing techniques to obtain binary silhouette image sequences that were 256 × 256 pixels in size. Table 2 illustrates the quantities of action sequences in both the RIDS and CIDS datasets.

### 3.4. Building and Optimizing the Model

**Figure 3:**
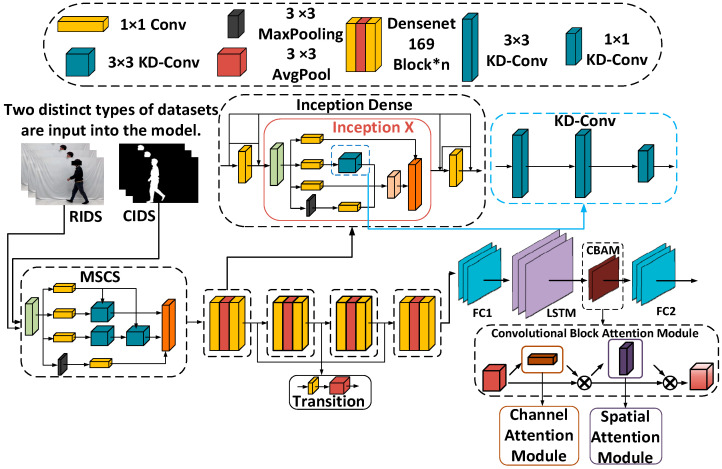
The structure of the MSCS–DenseNet–LSTM model.

Figure 3 shows the overall structure of the anomalous behavior recognition algorithm based on the improved spatiotemporal network proposed in this paper.

(1) Preprocess various video data to obtain RGB image sequences and contour image sequences, which are used as inputs to the network model.
(2) Convolutional network section: Firstly, replace the 7 × 7 convolution in the initial convolution layer of the original DenseNet169 model with a self-designed MSCS convolution module to construct a multi-scale convolution structure. This helps the model capture features at different levels of the image while increasing network width and adaptiveness to scale. Then, replace the BN + ReLU + 3 × 3 Conv structure in the DenseNet Block module with a BN + Leaky ReLU + 1 × 1 Conv + Inception X + 1 × 1 Conv structure, known as the Inception Dense structure. Replace the 3 × 3 convolution in the self-designed Inception X module with three layers of dilated convolutions with a rate of 2, which increases the receptive field while reducing the number of model parameters, decreasing complexity, and improving computational efficiency. Finally, conduct continuous multi-layer feature fusion in the DenseNet Block module before inputting to the fully connected layer.
(3) Temporal network section: The temporal network consists of two LSTM (Long Short-Term Memory) layers, a CBAM (Convolutional Block Attention Module), and a fully connected layer. With the introduction of the CBAM, dynamically adjusting attention weights and obtaining specific channel and spatial information helps effectively extract key features in the image through information fusion, thereby enhancing the model’s performance in visual tasks.
(4) Overall network structure: By performing tensor addition on spatial features extracted by the convolutional network and the spatiotemporal features extracted by the temporal network, the fusion of spatiotemporal features is achieved, compensating for the loss of spatial feature information and enabling the identification of abnormal behaviors through the fused spatiotemporal features.

#### 3.4.1. Construction of Convolutional Network—DenseNet

**Figure 4:**
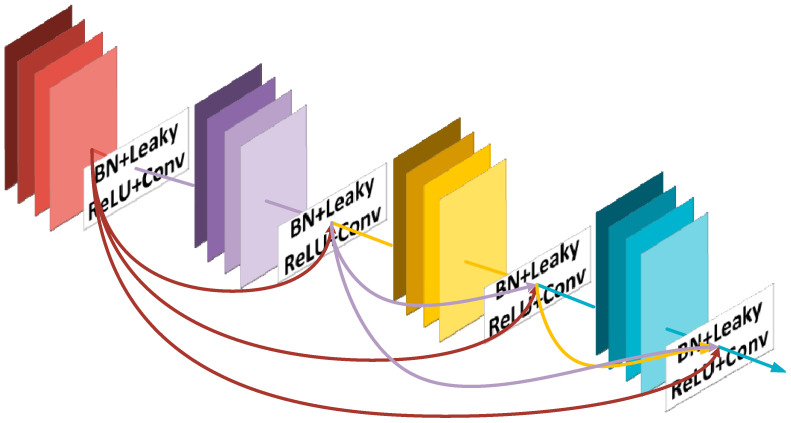
The DenseNet network architecture.

The convolutional layer is the core component of convolutional neural networks in the field of image recognition. In theory, by stacking a large number of convolutional layers, more image features can be extracted, thereby improving the efficiency of the model. However, with an increase in network structure, the stacking of a large number of convolutional layers can lead to a vanishing gradient and degradation problem. The emergence of DenseNet addresses this issue, with its feature sharing and arbitrary interconnection between layers. A DenseNet primarily consists of DenseNet Blocks and Transition Layers. The outputs of all previous DenseNet Block modules are concatenated with the current DenseNet Block module, generating a new feature map that is then input to the next layer of the DenseNet Block module. This allows the shallow features of the image to be repeated and effectively utilized, to some extent solving the vanishing gradient problem [31]. The Transition Layer module is used to connect multiple DenseNet Blocks, consisting of 1 × 1 convolutions and 2 × 2 average pooling, to compress the input of the DenseNet Block and all extracted feature information, reducing the size and dimension of the feature map, consequently reducing the model’s depth to address overfitting issues [31]. Due to the dense connections in the DenseNet network, the DenseNet169 model, with a deeper and wider network structure, is used in this study for abnormal behavior recognition. It is less affected by overfitting and vanishing gradient issues, and has more layers and parameters, effectively capturing complex features and adapting to more complex structures, thereby improving model performance and robustness [32]. Figure 4 illustrates the network structure of the DenseNet model.

DenseNet can be represented by the following formula:

(1) $$y_{i}=Z_{i}(x_{i})=Z_{i}([y_{0},y_{1},y_{2},...,y_{i-2,}y_{i-1}])$$

where $Z_{i}$(∙) is a non-linear transformation function, consisting of three parts: convolution, activation function, and batch normalization; and $\left[y_{0},y_{1},y_{2},...,y_{i-1}\right]$ describes the connectivity effect between DenseNet Block modules. This connection links the output of the previous layer *i* − 1 to a certain dimension of the next layer.

#### 3.4.2. Multi-Scale Convolutional Structure

**Figure 5:**
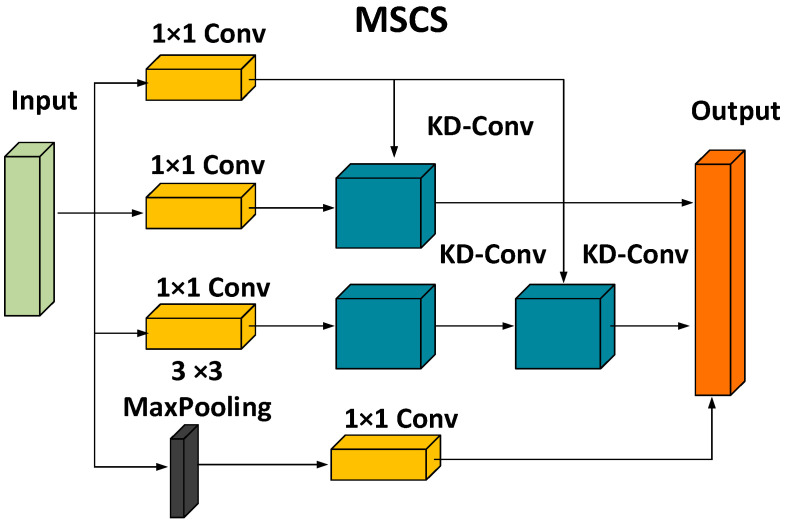
Multi-scale convolutional subsampling module.

The specific structure of the multi-scale convolution module constructed in this article is shown in Figure 5. This structure is a parallel structure that acquires feature map information at different scales through convolutional layers of different scale sizes, ultimately merging feature information from different scales to obtain more in-depth and rich behavioral features. Placing it at the initial position of the model structure not only addresses the issue of the insufficient feature extraction capability of the initial convolution module but also improves the model’s depth of exploration and feature accuracy. The presence of the parallel structure also reduces the computational complexity of the model, increasing the model’s computational efficiency.

Using the Multi-Scale Convolutional Structure (MSCS) for the initial convolution calculation mainly involves the following four convolution processes:

(1) The first convolution uses a regular 1 × 1 convolution to perform convolution calculations on the extracted feature map. Using a 1 × 1 convolution kernel helps alleviate the issue of increased model parameters caused by convolutions at different scales.
(2) In the second and third convolution operations, after passing through a 1 × 1 convolutional filter, the feature map is then fed into a layer of 3 × 3 convolution and two layers of consecutive 3 × 3 convolution kernels, respectively. The first 3 × 3 convolution operation captures low-level features in the original input data, while the second 3 × 3 convolution integrates these low-level features into high-level features, helping the model gradually learn higher-order abstract complex features, improving the model’s generalization ability, and addressing the issues of vanishing and exploding gradients.
(3) In the final convolution operation, after passing through a 3 × 3 max pooling layer, the input is then fed into a 1 × 1 convolution layer. The max pooling layer effectively preserves the main features in the image and reduces the spatial scale of the feature map to achieve dimensionality reduction, aiding the model in reducing computational complexity and improving computational efficiency.
(4) Although the feature extraction at each layer contributes to the improvement of model computational efficiency and generalization ability, without information exchange between layers, it reduces the network’s feature extraction capability. To address this issue, using Channel Shuffle [33], which has a lower computational cost and complexity, helps facilitate the flow of feature information between layers. Adding a batch normalization (BN) layer and Leaky ReLU activation function after each layer not only improves model performance and stability but also solves the issue of “neuron death”.

#### 3.4.3. Improvement of DenseNet Block

**Figure 6:**
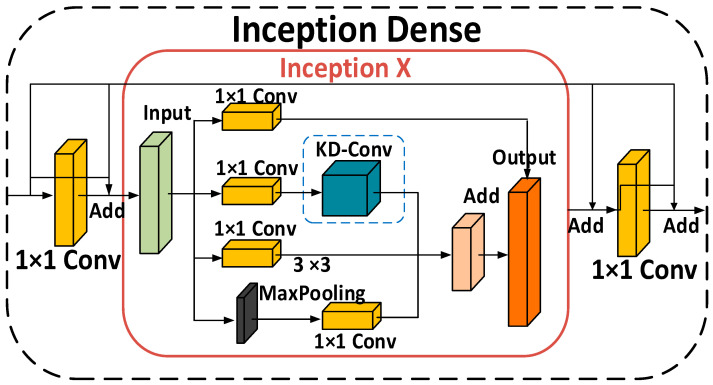
Structure diagram of Inception Dense.

In abnormal behavior recognition tasks in the human body, when the body performs various actions, the body’s structure will take different forms and different action amplitudes. Therefore, abnormal behavior recognition should be performed at multiple scales. The DenseNet Block structure consists of a BN + ReLU + 3 × 3 Conv structure, which can only extract features at a fixed scale and cannot meet the needs of multi-scale information extraction. In order to better capture the contextual multi-scale hierarchical information of feature maps, we chose to use a BN + Leaky ReLU + 1 × 1 Conv + Inception X + 1 × 1 Conv structure, naming it the Inception Dense structure. The structural diagram is shown in Figure 6.

The Inception Dense structure mainly performs the following convolution operations:

(1) In Inception Dense, the BN + Leaky ReLU structure is used before convolution. BN can normalize the data input to the Inception Dense structure, and Leaky ReLU activation function can solve the problem of “neuron death” caused by ReLU when the input is less than 0.
(2) Adding a 1 × 1 convolution before the Inception X structure can increase the channel number of the normalized feature map, linearly combine the feature map, introduce more diversity and expressive power, and help the Inception X structure to capture more abstract and complex features. Adding a layer of 1 × 1 convolution after the Inception X structure can reduce the dimensions of the feature map, decrease the number of model parameters, and improve the computational efficiency of the model.
(3) The self-built Inception X consists of four layers: (1) The first layer of convolution performs calculations using regular 1 × 1 convolutions. The resulting feature map, fused with the subsequent three layers, not only contains the shallow features of the original data but also includes deep features fused from the subsequent three layers, solving the problem of lack of information exchange between layers, enhancing the model’s computational efficiency and stability. (2) The second layer structure first undergoes a layer of 1 × 1 convolution before entering three continuous layers of 3 × 3 atrous convolutions. By gradually increasing the dilation rate, it can ensure the same receptive field while increasing the effective field of view of the convolutional kernel, thereby helping the model capture richer deep multi-scale feature information. (3) The last two layers consist of a 1 × 1 convolution layer and a 3 × 3 max pooling layer, where the 1 × 1 convolution effectively preserves the shallow features of the original data, and the 3 × 3 max pooling layer reduces the spatial scale of the feature map, improving the model’s computational efficiency. (4) The features from the last three layers are fused, containing the original data features processed by the 1 × 1 convolution, the richer deep multi-scale feature information obtained after the continuous atrous convolution layers, and the primary features of the original image after the 3 × 3 max pooling layer. After feature fusion, convolution kernels of different scales capture features at different levels, avoiding information loss in a single channel, and allowing the network to better adapt to data information and structures of different scales.

#### 3.4.4. Construction of Temporal Network—LSTM

**Figure 7:**
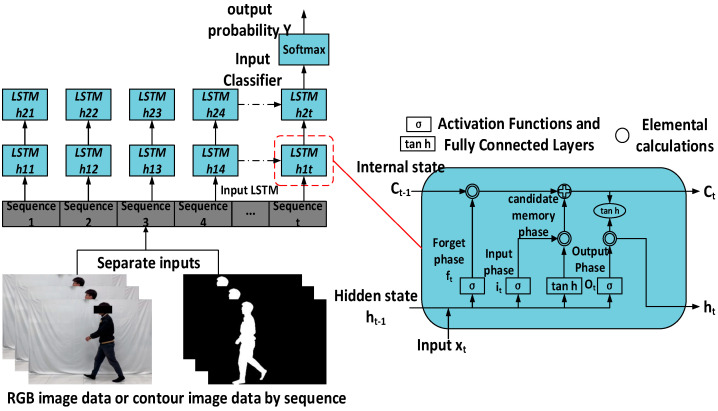
Dual-layer LSTM architecture.

Long Short-Term Memory Networks (LSTMs) are a special type of RNN that are particularly suitable for processing sequential data [34]. This paper constructs a two-layer LSTM network structure for the deep exploration of human behavioral temporal features, as shown in Figure 7. LSTM mainly consists of a forget phase, input phase, and output phase, and the internal operations are mainly divided into three stages: forget, memorize, and output. The formulas for each process are as follows:

Forget phase: Use the forget gate to implement the forgetting of long-term memory.

(2) $$f_{t}=\sigma (W_{xf}\cdot x_{i}+W_{hf}\cdot h_{t-1}+b_{f})$$

Memory phase: feature extraction of the input information and storage of important information in the internal state to complete the update of long-term memory.

(3) $$i_{t}=\sigma (W_{xf}\cdot x_{t}+W_{hi}\cdot h_{t-1}+b_{i})$$

Output phase: uses an output gate to select a valid output memory from the internal state.

(4) $$o_{t}=\sigma (W_{xo}\cdot x_{t}+W_{ho}\cdot h_{t-1}+b_{o})$$

(5) $$h_{t}=o_{t}⊗\tan h(C_{t})$$

where *f_t_*, *i_t_*, and *o_t_* denote the forgetting, input, and output gates, respectively, and *h_t_* is the carrier of short-term memory at moment *t*; *W* in Equations (2)–(4) represents the weights of the memory states of the three gates, and *b* represents the offsets of the memory states of the three gates.

#### 3.4.5. Convolutional Block Attention Module

For a temporal network, both the short-term and long-term temporal features of the data are indispensable. Although the LSTM network structure captures the long- and short-term temporal features of the data, since abnormal human behavior is a continuous process, not a single-frame image, it requires a more focused acquisition of the long-term temporal features of the video. Therefore, this paper adopts the CBAM attention mechanism to further focus on long-term temporal features [35].

**Figure 8:**
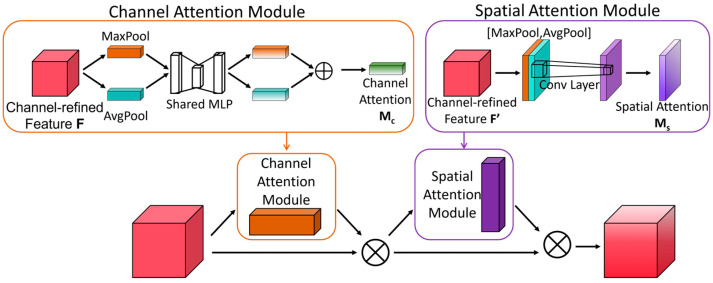
Convolutional Block Attention Module.

In this article, the CBAM attention mechanism is introduced after the LSTM network, with its structure shown in Figure 8. LSTM can effectively extract temporal information from the image sequence of human behavior, while CBAM can help the model capture global dependencies by dynamically adjusting the weights of each channel, suppressing unimportant features, and enhancing the network’s ability to extract long-term temporal features. The temporal information obtained by LSTM is combined with the global dependencies captured by CBAM, forming a more comprehensive and robust representation of human behavioral actions.

## 4. Experiment and Result Analysis

This experiment was based on the Linux operating system, Intel(**^®^**) Core(™) i9-7900X 3.30 GHz CPU, GPU GeForce RTX 3090, Python version 3.8.4, with the deep learning framework Pytorch. The parallel computing framework and version used were CUDA 11.4, and the development environment used was Pycharm.

The RIDS and CIDS datasets were randomly divided into training and test sets in proportion, with 80% of the data used for model training and 20% for model testing. The model optimizer used was Adam, and the loss function used was cross-entropy. The learning rate was set to 0.0005, the batch size to 8, and the epochs to 600. To prevent overfitting, dropout was set to 0.4.

### 4.1. Evaluation Indicators

To further validate the model’s effectiveness, this study evaluates accuracy, sensitivity, specificity, and convergence speed as the evaluation criteria, with the following indicator formulas:

(6) $$Accuracy=\frac{1}{N}\sum_{i=1}^{n}TP_{i}$$

(7) $$Sensitivity=\frac{1}{N}\sum_{i=1}^{n}\frac{TP_{i}}{TP_{i}+FN_{i}}$$

(8) $$Specificity=\frac{1}{N}\sum_{i=1}^{n}\frac{TN_{i}}{TN_{i}+FP_{i}}$$

Among them, *TP*, *TN*, *FP*, and *FN* represent true positive, true negative, false positive, and false negative, respectively. *N* is the total number of samples, *i* is the ith class, and *n* is the number of action categories. The convergence speed of the model can be judged by the number of epochs required for the model to converge.

### 4.2. Experimental Comparison of Self-Built Datasets

To compare the advantages and disadvantages of self-built and public datasets on the MSCS–DenseNet–LSTM model, the following sets of comparative experiments were designed, and the experimental results are shown in Table 3. From Table 3, it can be seen that the self-built CIDS and RIDS datasets in this paper outperformed the four groups of public datasets in all indicators. Compared with CIDS, the recognition effect of RIDS is better because RGB images contain rich color and texture information, which help the model more accurately capture subtle changes and features in human body movements. Therefore, all subsequent experiments in this paper choose to use the RIDS dataset as a comparative reference.

### 4.3. Ablation Study

This paper designs ablation experiments to verify the performance improvement of each step in the MSCS–DenseNet–LSTM model. This ablation experiment focuses on the original model, the introduction of the MSCS and Inception X modules, the design of the Inception Dense structure, the Kd-Conv replacement, and the addition of the CBAM attention mechanism. Table 4 shows the design of six sets of experiments (labeled 1–6).

Table 5 shows the comparative results of the ablation experiments. Experiment 2 introduced the MSCS module to the original model, increasing the accuracy, sensitivity, and specificity by 4.27%, 3.94%, and 4.17%, respectively. This indicates that the introduction of MSCS effectively addressed the initial problem of insufficient convolutional feature extraction capability, while obtaining deep and rich multi-scale behavioral features and resolving the lack of feature information exchanged between branches through channel shuffling. It also reduced the model’s computational cost and complexity significantly, enhancing the model’s convergence efficiency. Experiments 3 and 4 introduced the Inception X module and the Inception Dense structure, respectively. It is evident that just incorporating the Inception X module greatly improved the model’s performance, showing that the Inception X structure effectively addressed the inability of the Dense Block structure to extract multi-scale information and enhanced the model’s generalization ability by fusing features from different branches to adapt to data information and structures of different scales. Compared to Experiment 2, Experiment 4 showed improvements of 5.86%, 6.16%, and 5.62% in each metric, and improvements of 2.81%, 2.91%, and 2.81% compared to Experiment 3, suggesting that replacing the Inception Dense structure assisted the Inception X structure in capturing more abstract and complex features while significantly enhancing the model’s computational efficiency. Experiment 5 replaced the 3 × 3 convolution in the Inception X structure with three layers of 3 × 3 atrous convolution. Compared to Experiment 4, each metric increased by 2.12%, 1.97%, and 2.25%, indicating that atrous convolution increased the effective field of view, aiding the Inception Dense structure in extracting richer and deeper multi-scale feature information while reducing the model’s computational parameters and speeding up convergence. Experiment 6 introduced the CBAM attention mechanism module in the LSTM layer. Compared to Experiment 5, there was a significant improvement in each metric, reaching peak values of 1.35%, 1.39%, and 1.22%. The introduction of CBAM helped the LSTM focus more on obtaining long-term information in image sequences, enhancing the model’s robustness and computational efficiency. Finally, compared to the original model, the MSCS–DenseNet–LSTM model proposed in this study increased the recognition accuracy by 13.60% and doubled the convergence speed, reaching peak model performance and demonstrating good robustness and stability.

### 4.4. Comparison Experiment of LSTM Layers

In order to ensure that the structure and parameters of the MCSC–DenseNet model remained unchanged, different layers of LSTM models were studied for their impact on recognition accuracy. The comparative results are shown in Table 6. It can be seen from Table 6 that when the number of LSTM layers was set to two, the model performs best in all performance indicators. Therefore, the number of LSTM layers was set to two.

### 4.5. Results of Model Comparison

As shown in Table 7, to verify the model’s performance, performance comparisons were made with other excellent models on the self-built dataset RIDS and the public dataset URFD. The recognition accuracy of MSCS–DenseNet–LSTM on RIDS and URFD reached 98.80% and 94.50%, respectively. In this experiment, all models were trained from scratch, but whether in RIDS or URFD, MSCS–DenseNet–LSTM always reached the highest recognition accuracy with the fastest convergence speed. In addition, compared to other models, the proposed model shows significant improvements in sensitivity, specificity, and convergence speed, indicating that the model not only ensures high recognition accuracy but also strengthens robustness and computational efficiency.

### 4.6. Model Parameter Comparison Experiment

This study aims to validate the proposed MSCS–DenseNet–LSTM model. It seeks to demonstrate that, while maintaining high accuracy, the model has fewer parameters compared to traditional models such as AlexNet, VGG19, DenseNet169, ResNet50, and other existing superior models. The comparative results are displayed in Table 8 below. The experimental data are sourced from the self-constructed dataset RIDS.

The data presented in Table 8 indicate that the proposed MSCS–DenseNet–LSTM model significantly reduces the number of parameters compared to traditional models such as AlexNet, VGG19, and ResNet50, with reductions of 48.72M, 131.29M, and 13.18M, respectively. Simultaneously, the model achieves improvements in recognition accuracy of 22.95%, 19.37%, and 15.33%. Notably, the MSCS–DenseNet–LSTM model builds upon the DenseNet169 framework by incorporating a dual-layer LSTM network structure and the CBAM attention mechanism. Remarkably, even with these added structures and modules, the model reduces parameter count by 1.77M while enhancing recognition accuracy by 13.6%. When compared to existing models such as CNN&VGG16 and RGB-MHI CNN, the parameter count is reduced by 132.72M and 20.37M, respectively, with corresponding accuracy improvements of 2.2% and 2.96%. These results demonstrate that the MSCS–DenseNet–LSTM model not only possesses a high recognition capability but also successfully decreases the parameter count through innovative designs like the Multi-Scale Convolution Module (MCSC) and the Inception Dense module, ensuring the rapid identification of anomalous behaviors.

### 4.7. Confusion Matrix

**Figure 9:**
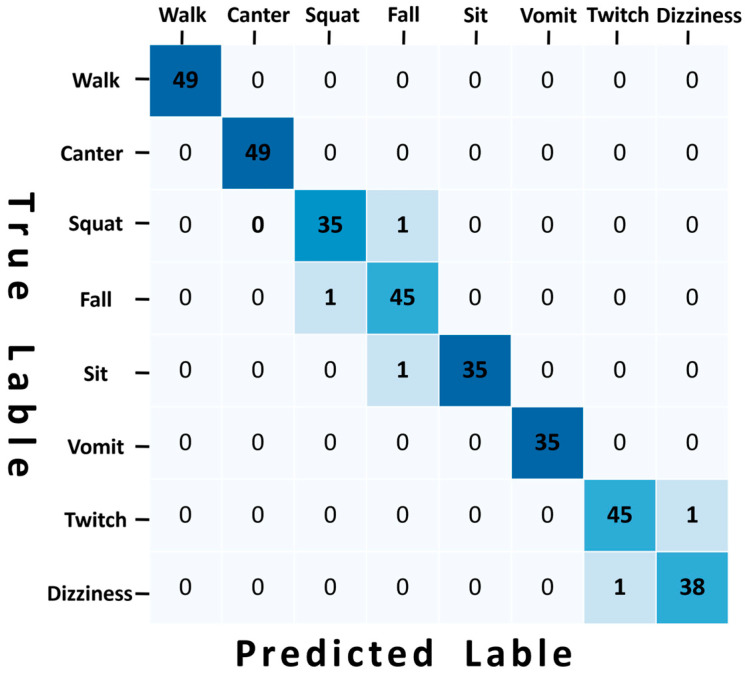
Confusion matrix.

The confusion matrix obtained by applying the MSCS–DenseNet–LSTM model to the self-built dataset RIDS is shown in Figure 9.

**Figure 10:**
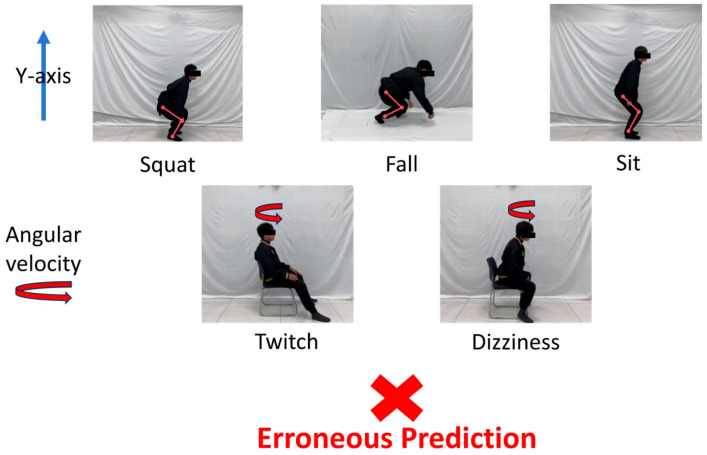
Error prediction of motion comparison graphs.

The confusion matrix indicates that a set of falling and squat actions were mistakenly identified as squats and falls, while a set of sitting actions were misclassified as falls. Additionally, movements characterized by twitching and dizziness were wrongly interpreted as dizziness and twitching. As illustrated in Figure 10, this misclassification may stem from the spatial displacement of limbs during squatting, sitting, and falling actions, all of which involve certain degrees of leg flexion, leading to model errors. Furthermore, the action amplitudes of twitching and dizziness fall within similar ranges, and the torso exhibits notable shaking, contributing to misidentification by the model. A comprehensive analysis of the confusion matrix and error prediction graphs reveals that extreme misclassifications predominantly occur between similar actions that may overlap in movement trends. Nonetheless, even under these circumstances, the occurrence rate of misclassification remains very low. In everyday life, only a few anomalous activities share identical motion trends, thereby demonstrating the practical applicability of the proposed MSCS–DenseNet–LSTM model in real-world scenarios.

**Figure 11:**
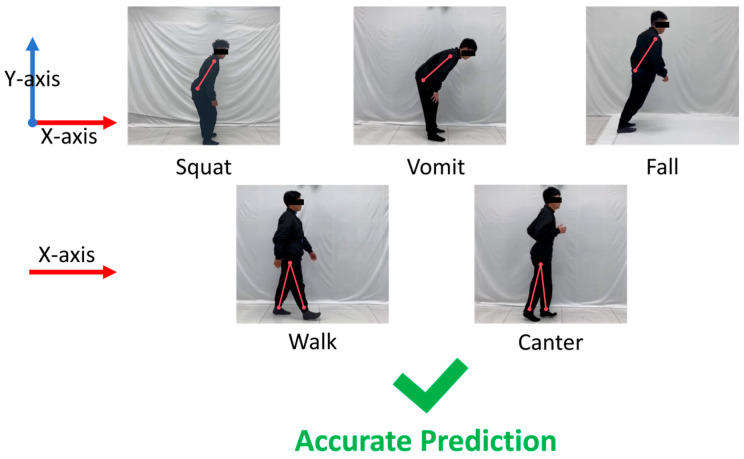
Accurate prediction of motion comparison graphs.

The confusion matrix indicates that the MSCS–DenseNet–LSTM model accurately predicts three behaviors with similar motion trends: squatting, vomiting, and falling. It also perfectly forecasts slow walking and jogging. Figure 11 reveals that squatting, vomiting, and falling exhibit some degree of similar curvature in the trunk region. In contrast, slow walking and jogging display comparable crossing angles in leg movements. Analyzing the confusion matrix alongside the correct prediction comparison confirms that this model precisely identifies actions with similarities and overlaps. Thus, this study demonstrates the practicality of the proposed MSCS–DenseNet–LSTM model in real-life scenarios.

The accuracy, sensitivity, and specificity of action recognition by the MSCS–DenseNet–LSTM model on the self-built dataset RIDS are shown in Table 9, with a recognition accuracy for various actions all above 95.74%, a sensitivity above 97.22%, and a specificity above 99.30%. The results above indicate that the model’s recognition of the eight types of actions is significant, especially in the recognition accuracy, sensitivity, and specificity of walking slowly, running slowly, and vomiting, all reaching 100%. This demonstrates that the model performs ideally in the recognition task of these three actions, achieving perfect and accurate recognition.

## 5. Conclusions

In this study, a new abnormal behavior recognition method called MSCS–DenseNet–LSTM is proposed to improve the model’s generalization ability while maintaining high accuracy. The model fully considers the spatiotemporal features of the data. By using the self-built RIDS dataset as the input, the proposed MSCS multi-scale convolution module merges shallow features from different branches to obtain richer deep-level features, effectively reducing data loss and accelerating model efficiency. An Inception Dense structure is designed to fuse features at various levels, unleashing the model’s parallel computing capabilities while ensuring high robustness. Additionally, a dual-layer LSTM structure with an added attention mechanism module is designed to effectively combine the temporal information in human action image sequences with the dependency relationship among frame rates focused by CBAM, forming a more robust human action recognition model structure with enhanced generalization ability. The introduction of this model significantly improves the speed and accuracy of abnormal action recognition, allowing users with mobility issues to receive more timely assistance in dangerous situations.

However, there are still many limitations in this study. Collecting authentic anomalous behavior data proved challenging. The data collection process involved participants wearing senior living experience suits in an ideal environment. This laboratory setting ensures high clarity and visibility, free from obstructions that might interfere with data collection. While this conditions highlight the optimal environment for model operation, it compromises the realism of the collected human data. Consequently, this limitation could affect practical applications. Additionally, the model’s performance on public datasets falls short, exhibiting poor recognition accuracy. This indicates that increasing the variety and volume of actions makes it difficult for the model to distinguish between behaviors with similar motions. Future research will explore integrating other anomalous behavior recognition methods, enhancing the efficiency of identifying diverse behaviors, and minimizing false positives. This will provide users with faster and more effective anomalous behavior recognition technology.

## Figures and Tables

**Table 1:** Detailed classification of anomalous behavioral actions.

Behavior Action	Detailed Classification
Falling	Falling forward while walking
	Falling backwards while walking
	Falling forward while rising
	Falling backwards while rising
Twitching	Lying spasms
	Lying tonic tic
	Sitting spasms
	Sitting tonic tic
Dizziness	Standing dizziness
	Sitting dizziness
Vomiting	Standing vomiting

**Table 2:** The quantity of RGB image and contour image sequences.

Action Types	Number of RGB Image Sequences	Number of Contour Image Sequences
Fall	230	230
Twitch	230	230
Dizziness	194	194
Vomit	176	176
Walk	246	246
Canter	246	246
Sit	180	180
Squat	180	180

**Table 3:** Comparison results of various datasets in the MSCS–DenseNet–LSTM model.

Datasets	Data Type	Accuracy (%)	Sensitivity (%)	Specificity (%)	Epoch
Enhancing ViBe Algorithm	Contour Image	97.30	97.20	97.88	480
ViBe	Contour Image	98.10	97.88	98.32	420
CIDS	Contour Image	98.30	98.28	98.38	400
FDD	RGB Image	96.70	96.32	96.30	450
URFD	RGB Image	97.33	97.00	97.89	400
RIDS	RGB Image	98.80	98.75	98.82	370

**Table 4:** Ablation study design.

Number	MCSC	Inception X	Inception Dense	KD-Conv	CBAM
1	×	×	×	×	×
2	√	×	×	×	×
3	√	√	×	×	×
4	√	√	√	×	×
5	√	√	√	√	×
**6**	**√**	**√**	**√**	**√**	**√**

**Table 5:** Experimental results of ablation study.

Number	Accuracy (%)	Sensitivity (%)	Specificity (%)	Epoch
1	85.20	85.29	85.56	780
2	89.47	89.23	89.73	650
3	92.52	92.48	92.54	560
4	95.33	95.39	95.35	480
5	97.45	97.36	97.60	410
**6**	**98.80**	**98.75**	**98.82**	**370**

**Table 6:** The accuracy of MSCS–DenseNet–LSTM at different LSTM layer depths.

Number of LSTM Layers	Accuracy (%)	Sensitivity (%)	Specificity (%)	Epoch
1	98.00	97.89	98.12	370
**2**	**98.80**	**98.75**	**98.82**	**370**
3	98.25	98.10	98.34	450

**Table 7:** Comparison of performance with other existing excellent models.

Models	Dataset	Accuracy (%)	Sensitivity (%)	Specificity (%)	Epoch
MobileVGG [36]	Self-built dataset (RIDS)	98.00	98.10	98.65	420
3D-CNN&VGG16 [37]		96.00	95.89	96.25	400
CBAM-IAM-CNN-BiLSTM [38]		98.12	97.79	98.73	380
**Proposed**		**98.80**	**98.75**	**98.82**	**370**
CBAM-IAM-CNN-BiLSTM [38]	Public dataset (URFD)	93.06	93.00	93.40	400
RGB-MHI CNN [39]		93.77	93.32	93.90	420
**Proposed**		**94.50**	**94.34**	**94.86**	**390**

**Table 8:** Comparison of model parameter experimental results.

Models	Params (M)	Accuracy (%)
AlexNet	61.10	75.85
VGG19	143.67	79.43
ResNet50	25.56	83.47
DenseNet169	14.15	85.20
CNN&VGG16	145.10	96.60
RGB-MHI CNN	32.75	95.84
**Proposed**	**12.38**	**98.80**

**Table 9:** Test results of the MSCS–DenseNet–LSTM model.

Action Type	Accuracy (%)	Sensitivity (%)	Specificity (%)
Walk	100	100	100
Canter	100	100	100
Squat	97.22	97.22	99.66
Fall	95.74	97.82	99.30
Sit	100	97.22	100
Vomit	100	100	100
Twitch	97.83	97.82	99.65
Dizziness	97.44	97.44	99.66

## Data Availability

The data presented in this study are available on request from the authors. The data are not publicly available due to the privacy concerns of subjects participating in the experiment.
